# Karyotypes of six spider species belonging to the families Gnaphosidae, Salticidae, Thomisidae, and Zodariidae (Araneae) from Turkey

**DOI:** 10.3897/CompCytogen.v8i2.6055

**Published:** 2014-05-16

**Authors:** Zübeyde Kumbıçak, Emel Ekiz, Serdar Çiçekli

**Affiliations:** 1 Department of Molecular Biology and Genetics, Faculty of Science and Art, Nevşehir Hacı Bektaş Veli University, 50300, Nevşehir, Turkey; 2 Department of Biology, Faculty of Science and Art, Nevşehir Hacı Bektaş Veli University, 50300, Nevşehir, Turkey

**Keywords:** Araneae, diploid number, sex chromosome system

## Abstract

In this study, the karyotypes of six spider species from Turkey belonging to the families Gnaphosidae, Salticidae, Thomisidae, and Zodariidae were analyzed. Male chromosomal features including diploid chromosome numbers and sex chromosome systems were determined as 2n=22, X_1_X_2_0 in *Drassyllus sur* Tuneva & Esyunin, 2003, *Nomisia exornata* (C. L. Koch, 1839), and *Nomisia orientalis* Dalmas, 1921; 2n=28, X_1_X_2_0 in *Sitticus caricis* (Westring, 1861); 2n=23, X0 in *Xysticus gallicus* Simon, 1875 and 2n=42, X_1_X_2_0 in *Pax islamita* (Simon, 1873), respectively. The chromosome morphology of all species was acrocentric. Data obtained contribute to knowledge of the karyotype evolution of araneomorphs.

## Introduction

Spiders are one of the most important animal groups, and contain approximately 44 500 species all around the world (Platnick 2014) and consists of three primary clades, namely Mesothelae, Mygalomorphae and Araneaomorphae, the last one being phylogenetically most derived and the largest group (Coddington and Levi 1991). Infraorder Araneaomorphae contains more than 41 000 species (Platnick 2014). Despite this diversity, only 771 species of spiders have been karyotyped so far (Araújo et al. 2014).

Entelegyne spiders form a very diversified clade of araneomorphs. Their karyotypes are characterized by a predominance of acrocentric chromosomes, X_1_X_2_0 sex chromosome system (Araújo et al. 2005), relatively low diploid chromosome numbers (ranges from 10 to 49, Kořínková and Král 2013), and chiasmatic meiosis (Kumbıçak 2010). Acrocentric karyotypes of entelegynes with lower chromosome numbers could be derived from ancestral acrocentric karyotypes by tandem fusions (Suzuki 1954) or by cycles of centric fusions and subsequent pericentric inversions (Kořínková and Král 2013). The latter hypothesis is supported by the fact that centric fusions are the most frequent source of chromosome polymorphism found in populations of entelegyne spiders (Kořínková and Král 2013).

In spiders, the X_1_X_2_0 system could be the ancestral sex chromosome determination as inferred from its presence in the most primitive recent spiders, namely the suborder Mesothelae and basal families of the infraorder Mygalomorphae (Suzuki 1954).

Salticidae, Thomisidae, Gnaphosidae, and Zodariidae are some of the largest families in the order Araneae (Platnick 2014). Despite the high diversity of these spider groups, cytogenetic data have been collected only in 223 species belonging to these clades (Araújo et al. 2014).

This study presents karyotypes of six species belonging to the genera *Drassyllus* Chamberlin, 1922 and *Nomisia* Dalmas, 1921 (Gnaphosidae), *Sitticus* Simon, 1901 (Salticidae), *Xysticus* C. L. Koch, 1835 (Thomisidae), and *Pax* Levy, 1990 (Zodariidae). Our study brings new data and fills some gaps in cytogenetics of these families.

## Material and methods

**Material**: Spiders were collected in Mediterranean, Southeast and Central Anatolia (Turkey) during the year 2012. Collection data of particular species (localities including their coordinates, dates of collection, number of individuals studied) are listed in Table 1. Voucher specimens were deposited in the collection of Department of Molecular Biology and Genetics, Art and Science Faculty, Nevşehir Hacı Bektaş Veli University (Nevşehir, Turkey). The identification of spiders was made by O. Seyyar (Department of Biology, Art and Science Faculty, Niğde University, Niğde, Turkey).

**Table:** **Table 1.** Material used for chromosome analysis.

**Family**	**Species**	**Locality**	**Coordinates**	**Date of Collection**	**Number of Individuals Studied**
Gnaphosidae	*Drassyllus sur* Tuneva & Esyunin, 2003	Gaziantep, Sakçagözü	37°10'18"N, 36°55'39"E	04.04.2012	7♂
	*Nomisia exornata* (C. L. Koch, 1839)	Antalya, Aksu	36°55'30"N, 30°48'29"E	24.03.2012	11♂
	*Nomisia orientalis* Dalmas, 1921	Antalya, Gazipaşa	36°16'23"N, 32°17'33"E	24.03.2012	2♂
		Osmaniye, Düziçi	37°15'02"N, 36°26'36"E	21.05.2012	5♂
		Adıyaman, Kahta	37°48'46"N, 38°38'20"E	11.03.2012	4♂
		Gaziantep, Islahiye	37°01'21"N, 36°37'24"E	06.04.2012	9♂
Salticidae	*Sitticus caricis* (Westring, 1861)	Nevşehir, Göreme	38°38'44"N, 34°50'06"E	10.05.2012	8♂
		Nevşehir, Zelve	38°40'16"N, 34°51'43"E	27.06.2012	3♂
Thomisidae	*Xysticus gallicus* Simon, 1875	Adana, Çamalan	37°19'12"N, 34°36'28"E	12.04.2012	6♂
		Mersin, Bozyazı	36°06'04"N, 32°58'38"E	15.04.2012	2♂
		Mersin, Aydıncık	36°08'36"N, 33°22'59"E	15.04.2012	3♂
Zodariidae	*Pax islamita* (Simon, 1873)	Osmaniye, Toprakkale	37°04'24"N, 36°08'42"E	09.06.2012	5♂

**Chromosome preparations and observation:** Slides for chromosome observations were made by the spreading technique of Traut (1976), with some modifications. This method consisted of three basic steps. First, the gonads were hypotonized in 0.075 M KCl for 12-15 min in room temperature (RT). Second, gonads were fixed in two batches of freshly prepared Carnoy fixative (ethanol: chloroform: glacial acetic acid; 6:3:1), first batch for 10 min and second one for 20 min (RT). Finally, a cell suspension was prepared from a piece of tissue in a drop of 60% acetic acid on a slide using a pair of tungsten needles. The slide was placed on a histological plate at 42 °C and the drop was evaporated by moving it with a tungsten needle. Slides were stained with 5% Giemsa in Sørensen phosphate buffer (pH=6.8) for 27 min (RT). Chromosome spreads were investigated an Olympus BX53 microscope and photographed using a DP26 digital camera (Olympus) using CELLSENS software (Olympus). Relative chromosome lengths (RCL) including standard deviations were calculated as a percentage of the total chromosome length of the diploid set including sex chromosomes (%TCL) from 10 mitotic metaphase plates for each species by CELLSENS software. Classification of chromosome morphology was based on the arm ratio (Levan et al. 1964).

## Results

### Gnaphosidae

**Figure 1.**
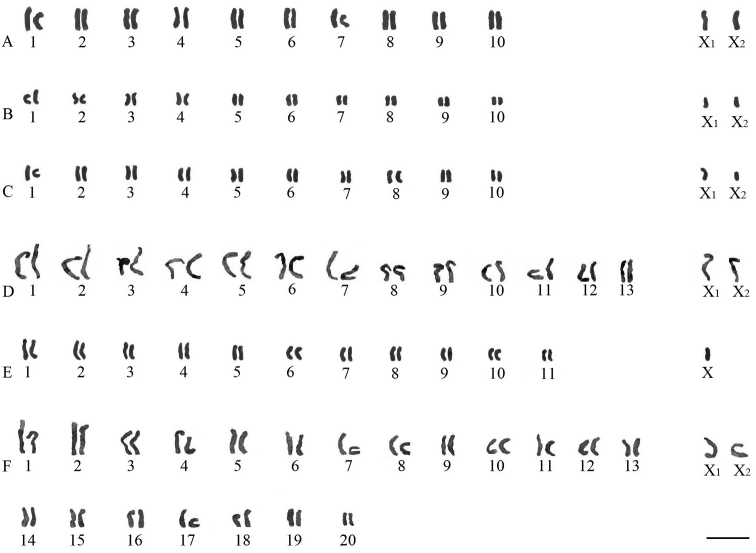
Karyotypes of species based on spermatogonial metaphases. **A** *Drassyllus sur*, 2n♂=22, X_1_X_2_0 **B** *Nomisia exornata*, 2n♂=22, X_1_X_2_0 **C** *Nomisia orientalis*, 2n♂=22, X_1_X_2_0 **D** *Sitticus caricis*, 2n♂=28, X_1_X_2_0 **E** *Xysticus gallicus*, 2n♂=23, X0 **F** *Pax islamita* 2n♂=42, X_1_X_2_0. Bar=10 µm.

The chromosomes of *Drassyllus sur* Tuneva & Esyunin, 2003 (2n♂=22) were acrocentric. The sex chromosome system was formed by chromosomes X_1_ and X_2_ which were medium-sized elements (Fig. 1A). The autosome pairs decreased gradually in size. Length of autosome pairs decreased from 9.74±0.29% to 6.89±0.12% of TCL. Relative length of X_1_ and X_2_ was 8.45±0.06% and 7.57±0.17% of the diploid set, respectively.

**Figure 2.**
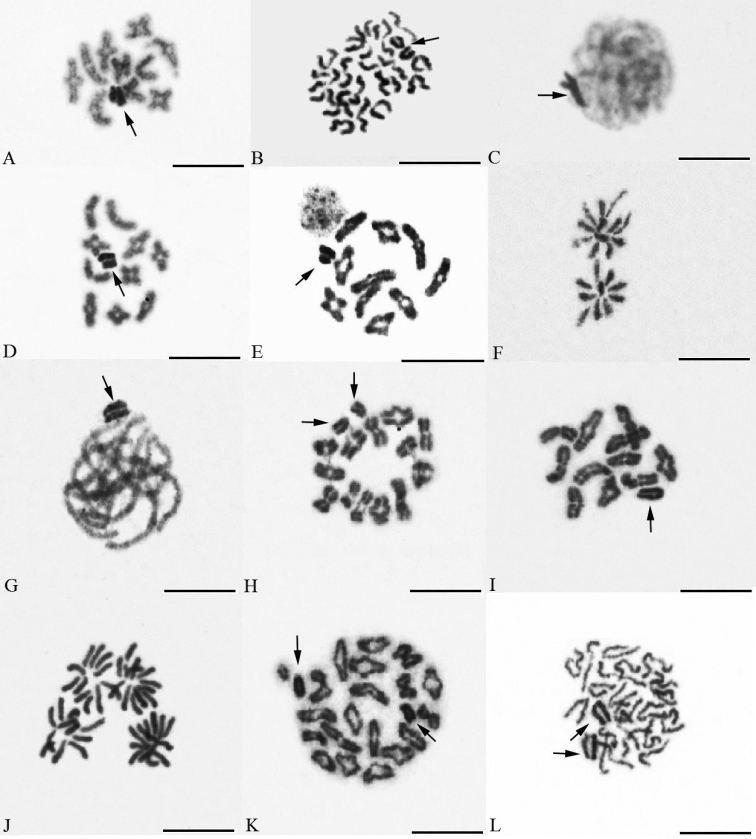
Meiosis of gnaphosid, salticid, thomisid and zodariid males. *Drassyllus sur* **A** diplotene **B** metaphase II, *Nomisia exornata* **C** early pachytene **D** diakinesis, *Nomisia orientalis* **E** diakinesis **F** part of anaphase II showing one plate with 10 chromosomes and another plate with 12 chromosomes, *Sitticus caricis* **G** pachytene **H** diplotene, *Xysticus gallicus* **I** diakinesis **J** anaphase II, *Pax islamita* **K** diakinesis **L** half of metaphase II (arrows indicate sex chromosomes). Bar=10 µm.

There were 10 autosomal bivalents and two sex chromosomes at diplotene (Fig. 2A). Sex chromosomes were positively heteropycnotic from leptotene to metaphase II (Fig. 2B).

The karyotype of *Nomisia exornata* (C. L. Koch, 1839) (Fig. 1B) (2n♂=22, X_1_X_2_0) was acrocentric. Autosome pairs decreased gradually in size from 10.3±0.21% to 5.85±0.17% of TCL. Relative length of X_1_ and X_2_ were 7.46±0.13% and 6.65±0.08% of TCL, respectively.

The autosomes of *Nomisia orientalis* Dalmas, 1921 (Fig. 1C) (2n♂=22, X_1_X_2_0) was acrocentric. RCL of autosome pairs were decreased gradually from 10.61±0.24% to 6.62±0.19% of TCL. The gonosomes X_1_ (7.91±0.12% of TCL) and X_2_ (6.10±0.07% of TCL) showed acrocentric morphology.

The sex chromosomes were positively heteropycnotic from leptotene to diakinesis in both *Nomisia* species studied. Plates consisted of 10 autosomal bivalents and two univalent sex chromosomes from pachytene to metaphase I (Fig. 2C–E). At meiotic anaphases, 10 chromosomes segregated to one pole and 12 chromosomes to another pole (Fig. 2F).

### Salticidae

The autosomes of *Sitticus caricis* (Westring, 1861) (2n♂=28, X_1_X_2_0) were acrocentric. RCL decreased gradually from 8.47±0.42% to 5.04±0.16% of TCL (Fig. 1D). The sex chromosomes X_1_ (7.33 ±0.51% of TCL) and X_2_ (6.72±0.38% of TCL) were medium sized in comparison with the autosomes.

Leptotene, zygotene, and pachytene nuclei included a positively heteropycnotic sex chromosome body that was located at the periphery of the nucleus (Fig. 2G). At late prophase I (i.e. diplotene and diakinesis), 13 autosomal bivalents and two univalent sex chromosomes were determined (Fig. 2H).

### Thomisidae

The chromosome set of *Xysticus gallicus* Simon, 1875 (2n♂=23, X0) contained 11 acrocentric pairs and a small X chromosome (Fig. 1E). Autosome pairs decreased gradually in size from 10.28±0.62% to 6.46±0.39% of TCL. Relative length of X chromosome was 6.77±0.46% of TCL. This chromosome was longer than the smallest autosome pair.

From leptotene to diakinesis, X chromosome was formed by an intensively stained material. Diakinetic plates exhibit 11 autosomal bivalents (Fig. 2I). At metaphase II and anaphase II, X chromosome was isopycnotic with autosomes (Fig. 2J).

### Zodariidae

The karyotype of *Pax islamita* (Simon, 1873) consisted of acrocentric chromosomes; the diploid number was 42 (Fig. 1F). Autosome pairs exhibited a gradual decrease of relative lengths from 6.42±0.58 to 3.31±0.24% of TCL. This species showed X_1_X_2_0 sex chromosome system. The acrocentric gonosomes showed similar size. Their relative lengths were 5.92±0.66% and 5.37±0.18% of TCL, respectively.

From beginning of pachytene to metaphase I, plates consisted of 20 autosomal bivalents and two not associated sex chromosomes on the periphery of nucleus (Fig. 2K). Sex chromosomes were positively heteropycnotic during prophase and metaphase II. Metaphases II consisted of 20 or 22 chromosomes, respectively. Metaphases II with 22 chromosomes contained two X chromosomes (Fig. 2L).

## Discussion

Karyotypes of 771 spider species from 277 genera are known at present (Araújo et al. 2014). Diploid chromosome numbers of spiders range from 7 (Suzuki 1954) to 128 (Král et al. 2013). Entelegynae araneomorphs exhibit lower diploid numbers and mostly monoarmed chromosomes when compared with the predominantly high chromosome numbers and biarmed chromosomes of mygalomorphs (Kořínková and Král 2013). The sex chromosome system of most entelegynes is X_1_X_2_0 type. This system is supposed to be the ancestral form in spiders. It was found in more than 77% of spiders (Araújo et al. 2005).

So far, chromosome numbers have been established for 38 species of gnaphosid spiders. The majority of species (33 in a total) have 2n♂=22 including X_1_X_2_0 sex chromosome system (Araújo et al. 2014). Cytogenetics of *Drassyllus* is still not adequately explored. However, two karyotypes of *Drassyllus* have been published: *Drassyllus pumilus* (C.L. Koch, 1839) (2n♂=22, X_1_X_2_0) (Kumbıçak et al. 2009) and *Drassyllus praeficus* (L. Koch, 1866) (2n♂=22, X_1_X_2_0) (Kumbıçak et al. 2013). The same karyotype was found in *Drassyllus sur* (this study). Karyotypes of the *Nomisia* species analyzed show also the same karyotype with 2n♂=22 and X_1_X_2_0 sex chromosome system (Gorlova et al. 1997, Kumbıçak et al. 2011, this study). *Drassyllus* and *Nomisia* belong to different subfamilies (Zelotinae and Gnaphosinae, respectively) (Ubick 2005, Seyyar et al. 2009). With one exception (*Urozelotes rusticus*, (L. Koch, 1872) Srivastava and Shukla 1986), all members of these subfamilies karyotyped so far presents 2n♂=22, X_1_X_2_0 (Araújo et al. 2014), confirming the homogeneity of chromosome numbers, morphology and sex chromosomes systems in the family Gnaphosidae.

Male diploid numbers in salticids vary from 2n=14 in *Menemerus illigeri* (Audouin, 1826) (Gorlova et al. 1997) to 2n=28 in most salticids (109 in a total, Araújo et al. 2014). According to the previous studies, salticids exhibit considerable diversity of the sex chromosome systems (X0, X_1_X_2_0, X_1_X_2_X_3_0, and X_1_X_2_X_3_Y). 12 male karyotypes has been found in salticids, namely: 29, X_1_X_2_X_3_0; 28, X_1_X_2_0; 27, X0; 27, X_1_X_2_Y; 26, X_1_X_2_X_3_Y; 26, X_1_X_2_0; 25, X_1_X_2_Y; 25, X0; 23, X0; 22, X_1_X_2_0; 21, X0 and 14, X_1_X_2_0 (Araújo et al. 2014). Ancestral karyotype of salticids is probably formed by 28 chromosomes including X_1_X_2_0 system (Maddison and Leduc-Robert 2013). Our results showed 2n♂=28, X_1_X_2_0 in *Sitticus caricis*. This chromosome number as well as the acrocentric chromosome morphology is the same as found in *Sitticus* species studied so far, namely *Sitticus littoralis* (Hahn, 1832) (Suzuki 1954) and *Sitticus terebratus* (Clerck, 1757) (Hackman 1948). *Sitticus* is the only genus belonging to Amycoida clade (Maddison and Hedin 2003) karyotyped so far. So, the current knowledge is not sufficient to explain the karyotype evolution of this clade, therefore new studies on the other amycoids are needed.

The male karyotype of *Xysticus gallicus* displays the general pattern described for most Thomisidae: a diploid chromosome number 23 and X0 sex chromosome system including acrocentric sex chromosome. All *Xysticus* species analyzed so far present this karyotype with exception of *Xysticus triguttatus* Keyserling 1880 (Painter 1914). According to the phylogeny of Benjamin et al. (2008), *Xysticus* is sister group to *Coriarachne* Thorell, 1870, that also presents 2n=23, X0 (*Coriarachne fulvipes* Karsch, 1879; Suzuki 1952). There are two hypotheses explaining the origin of acrocentric X0 sex chromosome system in spiders. According to Datta and Chatterjee (1989, 1992) the acrocentric X chromosome can be derived by centric fusion of the X_1_ and X_2_ chromosomes, followed by pericentric inversions. Also, the acrocentric X chromosome could have originated from tandem fusion between acrocentric X_1_ and X_2_ chromosomes (Pekár and Král 2001).

Our study represented a diploid number of 42 acrocentric chromosomes and X_1_X_2_0 system in *Pax islamita*. This finding is compatible with the results reported by Král et al. (2011). However, these authors have also found heterozygotes for autosomal centric fusion (2n♂=41, X_1_X_2_0) in addition to the standard individuals. Furthermore, they revealed different pattern of sex chromosome behaviour at male germline of this species. According to their results, the gonosomes were recognised as early as spermatogonial prophase and prometaphase due to their precocious condensation, positive heteropycnosis, and association. However, our data showed the sex chromosomes were indistinguishable at mitotic prophase and prometaphase from autosomes. It was possible to recognise them from autosomes at the beginning of meiotic prophase only due to positive heteropycnosis.

In conclusion, our study described the karyotype features of five araneomorph spiders for the first time and confirms some findings of Král et al. 2011 for *Pax islamita*.
